# Downstream Signaling of Muscarinic M_4_ Receptors Is Regulated by Receptor Density and Cellular Environment

**DOI:** 10.1002/prp2.70123

**Published:** 2025-05-19

**Authors:** Madeleine Merz, Charlotte Playle, Monika Palchaudhuri, Oliver Hucke, Ulrike Gross, Daniel Ursu

**Affiliations:** ^1^ Boehringer Ingelheim Pharma GmbH & Co. KG Div. Research Germany Biberach an der Riss Germany

## Abstract

Multiple muscarinic M_4_ receptor modulators are currently advancing in clinical development for the treatment of positive symptoms in schizophrenia, including agonists and positive allosteric modulators. Considering the importance of comprehending M_4_ receptor pharmacology for these therapeutic applications, this study investigates M_4_ receptor signaling pathways upon activation by structurally diverse muscarinic agonists, exploring the role of receptor expression levels and cellular environment on downstream signaling. HEK‐293 cells and rat primary neurons expressing human M_4_ receptors were used to measure the kinetics of cAMP levels and compound effects on neuronal network activity. Receptor expression levels were controlled by a Tet‐On system and quantified using a radioactive binding assay. Our findings revealed that most agonists caused a concentration‐dependent reduction of cAMP levels (G_i/o_) at low concentrations, while inducing an increase in cAMP at higher concentrations (G_s_). A less prominent coupling via G_s_ was observed when receptor density in HEK‐293 cells was reduced. In the neuronal assay, most compounds showed consistent inhibition of neuronal activity. A distinct group of agonists displayed a specific profile, with no G_s_ coupling at high receptor density, partial activation at low receptor density, and low to no effects in the neuronal assay. This study provides a side‐by‐side comparison of the activity of structurally diverse M_4_ agonists and highlights compound‐specific activation of GPCR intracellular signaling pathways. The data offer new insights into M_4_ receptor pharmacology that may aid in the development of novel therapies for the treatment of psychiatric diseases.

## Graphical abstract

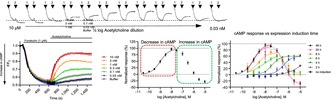

**Abbreviations**
AC: Adenylyl Cyclate
Bmax: maximum specific binding
BSA: bovine serum albumin
cAMP: Cyclic AMP
DIV: days in vitro
EC_50_
: effective concentration 50%
ECV: extracellular vestibule
EPAC: exchange proteins directly activated by cAMP
FCS: fetal calf serum
FLIPR: fluorescence imaging plate reader
GPCR: G protein coupled receptor
HBTS: HEPES buffered tyrode solution
HEK: human embryonic kidney
HEPES: 2‐(4‐(2‐Hydroxyethyl)‐1‐piperazinyl)‐ethansulfonacid
hM_4_
: human muscarinic acetylcholine receptor subtype 4
mAchR: muscarinic Acetylcholine Receptor
MOI: multiplicity of infection
MTP: microtiter plate
nAChR: nicotinic Acetylcholine Receptor
pEC_50_
: The negative logarithm of the EC_50_ value
PTX: pertussis toxin
rCN: rat cortical neuron
rH: relative humidity
RT: room temperature
SEM: standard error of the mean
Tet: tetracycline

## 1 Introduction

Muscarinic acetylcholine receptors (mAChRs) are G‐protein coupled receptors (GPCRs) activated by the neurotransmitter acetylcholine and found in the nervous system and other tissues, where they play an important role in regulating various physiological processes [1]. Five subtypes exist, with intracellular signaling pathways coupled to distinct types of G proteins: M_1_ , M_3_ , and M_5_ act preferentially through G_q_, while M_2_ and M_4_ use G_i_ proteins [2, 3, 4]. M_1_, M_2_, and M_3_ are widely distributed throughout the body, while M_4_ receptors are primarily found in the brain, specifically in the basal ganglia, hippocampus, and cerebral cortex. Additionally, M_5_ receptors are expressed in specific regions and cellular populations within the midbrain dopaminergic neurons. M_4_ receptors are believed to play a crucial role in regulating various physiological processes, being involved in learning and memory, motor control, temperature, and cardiovascular regulation [3]. Due to their high potential therapeutical significance, M_4_ receptors have gained significant interest in drug discovery as a potential target for the symptomatic treatment of various psychiatric and neurological disorders, including Schizophrenia, Parkinson's disease, and Alzheimer's disease, and hence multiple programs were initiated for finding selective drug candidates [4, 5, 6, 7].

In recent times, several drugs targeting the M_4_ receptor with varying selectivity profiles have demonstrated promising advancements in clinical development studies. These include the pan‐muscarinic agonist xanomeline developed by Karuna Therapeutics marketed in combination with trospium, a peripherally restricted muscarinic antagonist, as KarXT [8], a selective M_4_ agonist, NBI‐1117568, developed by Neurocrine Biosciences & Sosei Heptares [9], and the highly selective M_4_ positive allosteric modulator (PAM) Emraclidine developed by Cerevel Therapeutics [10]. Upon conclusion of two successful clinical trials, KarXT has been approved by the FDA in September 2024 as the first drug that provides a novel mechanism of action different from the classical dopamine receptor targeted therapies [11]. Subtype selectivity represents the critical property of these novel drugs which can influence not only the overall efficacy but also the adverse events profile. Because of the highly conserved composition of the orthosteric site across the subtypes of the mAChRs, it was suggested that the highest selectivity can be achieved only by targeting the allosteric site of the receptor [5, 6, 12]. Unfortunately, the former mode of action, PAM, has been invalidated recently in a failed clinical trial. The M_4_ PAM Emraclidine failed in a phase 2 study by not meeting its primary endpoint [13].

Typically, M_4_ receptors are G_i_ coupled receptors that signal through two main downstream intracellular pathways: the cyclic AMP (cAMP) pathway and the activation of G protein‐coupled inwardly rectifying potassium (GIRK) channels. The GTP‐bound G_αi_ subunit inhibits adenylyl cyclase, which effectively blocks cAMP production. On the other hand, the G_βγ_ subunits of G_i/o_ coupled GPCRs are known to interact with the GIRK channels present in the neuronal membrane, resulting in a negative shift in the membrane potential. This hyperpolarization and the reduction of cAMP levels ultimately lead to the inhibition of neuronal activity. It is expected that an agonist would modulate both pathways in a similar way, no compound being reported so far that showed biased signaling of M_4_ receptors in regard to the cAMP and GIRK pathways [14, 15, 16].

During the process of setting up cAMP cellular assays for investigating M_4_ receptors, we noticed changes in the canonical coupling of the receptor when activating it with the endogenous agonist acetylcholine. Specifically, a transition from the G_i_ inhibitory effect on cAMP (i.e., reduction of cAMP levels) to the G_s_ stimulatory effects (i.e., increase in cAMP levels) was observed, which exhibited a bell‐shaped concentration‐dependent pattern. Previous studies have shown that muscarinic M_4_ agonists are able to present biased signaling at the M_4_ receptor, activating different types of G proteins [17, 18, 19]. We decided to further investigate the initial observations by focusing on the impact of receptor expression on the cAMP modulation and on the GIRK signaling pathways. Therefore, a chemically diverse set of agonists, each exhibiting distinct selectivity levels at mAChRs, was profiled and their effects measured in a cAMP assay across a range of M_4_ receptor expression levels. Additionally, we expanded our investigation to explore M_4_ receptor pharmacology in a native environment by expressing M_4_ receptors in rat primary neurons using an AAV viral transduction system. For this part of the study, both cAMP dynamics and effects of agonists on spontaneous network neuronal activity were assessed. Overall, the findings presented in this study significantly strengthen our understanding of the mechanism of action of various muscarinic agonists at the M_4_ receptor. Additionally, our research demonstrates that different structural classes of agonists could activate distinct intracellular signaling pathways, suggesting the presence of biased signaling for this specific subtype of mAChRs, which further advances the previously established knowledge in the field.

## 2 Methods

### 2.1 Compounds

Acetylcholine was purchased from Sigma Aldrich (US), oxotremorine‐M and carbachol from Tocris (UK) and xanomeline, clozapine, compound‐5 PAM [20], example 76, compound‐6 agonist [21], example 3–3, isomer 2, and compound‐110 agonist [22] were synthesized at Boehringer Ingelheim, as well was MK‐6884, while [^3^H]‐MK‐6884 [23] was synthesized at Pharmaron (US). Given that compounds 5 and 6 have not previously been documented in literature and thus lack an established substance code, we have chosen to assign a simple numerical label to each of them. Retigabine was purchased from Tocris (UK), forskolin and pertussis toxin (PTX) from Sigma Aldrich (US). Compounds were dissolved in water or DMSO at stock concentrations of 10 mM or 100 mM and stored at −20°C. PTX was stored at 4°C at 0.1 mg/mL in water. Dilutions were prepared in the appropriate assay buffer.

### 2.2 HEK‐293 Human M_4_ Cell Culture

HEK293 FlpIn T‐Rex cells (Thermo Fisher Scientific, US) were used as the parental cell line and transfected with the human M_4_ (hM_4_)‐pcDNA5/FRT/TO and pOG44 plasmids (Geneart, Germany) using Lipofectamine 2000 (Invitrogen, US) for the generation of a stable HEK T‐Rex Tet‐On hM_4_ cell line. The gene sequence corresponding to the accession number NM_000741.2 for the hM_4_ receptor (gene name CHRM4) was used in this study. Cells were cultured in PDL‐coated flasks with RPMI 1640 Medium + GlutaMAX (Gibco, US) with 10% fetal calf serum (FCS; tetracycline tested, HyClone Laboratories, US), 15 μg/mL blasticidin S (Gibco, US) and 200 μg/mL hygromycin B (Invitrogen, US). Incubation parameters were 37°C, 5% CO_2_, and 95% relative humidity (rH).

### 2.3 Primary Neuron Culture

Primary cortical neurons (rCNs) were prepared from the brains of embryonic (E18) rats (Spraque‐Dawley, Janvier Labs, France) following the protocol described in detail in [24]. Dissociated neurons were plated on PDL‐coated 96‐well microtiter plates (MTPs) with a clear flat bottom (Corning, US) in serum‐free Neurobasal medium containing Glutamax and B27 supplement (Gibco). On day in vitro 1 (DIV1), virus transduction with 150 multiplicity of infection (MOI) AAV1/2‐hM_4_‐P2A‐mCherry was performed, combined with AAV‐EPAC1 transduction (2500 MOI) for neurons used in single‐cell cAMP imaging. Both virus stocks were produced at Boehringer Ingelheim. Partial medium exchange was executed on DIV7 for fluorescence imaging plate reader (FLIPR) experiments performed on DIV9 and DIV10, and on DIV4, DIV7, and DIV11 for single‐cell imaging experiments performed on DIV14 and DIV15.

### 2.4 cAMP Recordings in HEK293 hM_4_ Cells

After culturing HEK293 cells for 3–4 days without antibiotics, they were transferred at a density of 5 × 10^4^ cells/well into PDL‐coated 96‐well MTPs with a clear flat bottom 48 h prior to the recording. 2 h later, they were transduced with the green down cADDis cAMP sensor BacMam (#D0200G Montana Molecular, US) by addition of 4 × 108 viral genomes (VG)/mL cADDis BacMam and 0.3 mM Sodium Butyrate (final concentration). Dependent on the experimental protocol, M_4_ expression was induced with 0.5 μg/mL doxycycline (Sigma Aldrich, US) for various time intervals (between 2 h and 48 h) before the recordings or treated with 0.1 μg/mL PTX 24 h prior to the experiment.

FLIPR Tetra and Penta (Molecular Devices, US) instruments were used, which allow automated single or double compound addition with parallel recording of fluorescent signals (green fluorescence filter settings: excitation 470–495 nm, emission 515–575 nm). Cells were washed with HEPES buffered Tyrode solution (HBTS, Gibco, US) and incubated at room temperature (RT) for 20 min before FLIPR recordings were carried out. Test compounds were diluted in HBTS with a maximum DMSO concentration of 0.3% and added to the cells during recording. Signals were recorded in 5 s intervals (exposure time 0.5 s). Baseline signals were recorded for 1 min, changes of addition 1 for 10 min, and changes of addition 2 for another 15 min.

### 2.5 cAMP Recording in Primary Neurons

On DIV14 or DIV15, the medium of the rCNs expressing the hM_4_ receptor and Epac1 sensor was exchanged for HBTS assay buffer. After a minimum of 20 min, recordings were performed in a live cell imaging setup similar to the one described in [25]. In summary, the system was composed of an inverted fluorescence microscope (Axiovert S100, Zeiss, Germany) connected to a camera (Thorlabs, Germany) and a perfusion system. This setup enabled the wash‐in of compounds onto the cells within the field of view and facilitated continuous perfusion. We recorded the baseline for 1 min, followed by the addition of 3 μM forskolin for 3 min until a steady signal was achieved. During the subsequent addition of the compound (10 μM for 3 min), the forskolin level was maintained constant. We used a 405 nm LED for excitation and recorded the emission with a 515 nm long pass filter.

### 2.6 Recording of Spontaneous Ca^2+^ Oscillations in Primary Neurons

To record neuronal Ca^2+^ spontaneous oscillations, we employed a procedure similar to the one described in [24, 26]. In brief, measurements were conducted on DIV9 and DIV10, and the medium was replaced with HBTS buffer containing 4 μM of Fluo‐4 AM (Invitrogen, US). Neurons were incubated for 1 h at RT in the dark. Subsequently, the cells were washed with HBTS and incubated at RT in the dark for an additional 20 min. For FLIPR recordings, we used the green fluorescence filter settings (excitation 470–495 nm, emission 515–575 nm) and captured images every second (with a 0.5 s exposure). Each interval between compound additions (baseline, addition 1, addition 2) was recorded for a duration of 5 min.

### 2.7 Radioligand Binding Experiments

The expression of M_4_ receptors was induced in HEK293 cells with 0.5 μg/mL doxycycline at 48 h, 24 h, 8 h, 6 h, 4 h, and 2 h prior to the experiment. Cells were detached and resuspended in Receptor Binding Assay (RBA) Buffer (5 mM MgCl_2_, 1 mM CaCl_2_, 25 mM HEPES, pH 7.4; Sigma Aldrich, US) with 0.5% bovine serum albumin (BSA, Sigma Aldrich, US). For these experiments, the neurons were transduced with 150 MOI AAV1/2‐hM_4_‐P2A‐mCherry on DIV1. The optimum MOI was determined in a separate experiment (not described in this manuscript). On DIV 11, neurons were washed with Dulbecco's Balanced Salt Solution (DPBS; without Mg^2+^ and Ca^2+^) and detached with a cell scraper in RBA buffer containing 0.5% BSA. RBA buffer (to detect total binding) or nonradioactive MK‐6884 (to detect nonspecific binding), acetylcholine (100 μM) and radioligand [^3^H]‐MK‐6884 (0.0625–8 nM, 1:2 dilution steps) were mixed in a 96‐well MTP before the experiment was started by adding the respective cell suspension (HEK293: 5 × 10^4^ cells/well; rCN: 6 × 10^4^ cells/well). Plates were incubated on a shaker (IKA labs, Germany) for 1 h at RT. Afterwards, the assay was transferred on a 0.5% polyethylenimine treated GF/B Unifilter (Packard BioScience, US) by washing the assay plate with cold 0.9% NaCl (Sigma Aldrich, US) via Filtermate Harvester (Packard BioScience, US). Wells of the dried plate were filled with 50 μL MicroScint‐20 (Perkin Elmer, US) and after incubation for 1 h at RT in the dark, the plate was recorded in the Top Count NXT Imager (Perkin Elmer, US).

### 2.8 Data Analysis, Figure Preparation and Statistics

For analysis of the cAMP FLIPR experiments, kinetic fluorescence data (F) were divided by the signal of the baseline fluorescence (F_0_). ΔF/F_0_ gives the relative change in fluorescence, which was used to fit the agonist effect with the Graph Pad Prism's nonlinear regression model ([*Agonist*] vs. *response—Variable slope* [*four parameters*]):

$$Y=\text{Bottom}+\frac{\left(x^{\text{Hillslope}}\right)*\left(\mathrm{Top}-\text{Bottom}\right)}{x^{\text{Hillslope}}+{\mathrm{EC}}_{50}^{\;\text{Hillslope}}}$$

For analysis of the Ca^2+^ oscillation experiments, full‐time sequences were exported and single peaks larger than the threshold of 100 relative fluorescence units (RFU) were counted for each interval by using an Excel macro. Depending on the type of experiment, the oscillation ratios between addition 1 and baseline (addition 1/baseline) or between addition 2 and baseline (addition 2/baseline) were calculated and normalized to changes following the buffer addition = 100%. Curves were described with *([Inhibitor]* vs. *response—Variable slope (four parameters))*:

$$Y=\frac{\text{Bottom}+\left(\mathrm{Top}-\text{Bottom}\right)}{1+{\frac{{\mathrm{IC}}_{50}}{x}}^{\text{Hillslope}}}$$

Binding data were exported from Top Count NXT Imager. Specific binding was determined by transforming Y = A−B (A = total binding, B = unspecific binding) and then fitted by nonlinear regression *One site—Specific binding*:

$$Y=\frac{B_{\max}*X}{\left(\mathrm{Kd}+X\right)}$$

B_max_ was transformed from counts per minute (cpm) ± standard error of the mean (SEM) to binding sites/cell ± SEM via the GraphPad Radioactivity Calculator (https://www.graphpad.com/quickcalcs/radcalcform/ (15.11.2024); specific activity 83 Ci/mmole, counter efficiency 60%).

Figures were created with Prism (GraphPad), which was also applied for statistical analysis. For comparing the cAMP data in rCNs, the Brown‐Forsythe and Welch ANOVA tests have been used, followed by Dunnett's T3 multiple comparisons test with α of 0.05.

### 2.9 Molecular Modeling

Binding modes of clozapine and compound‐6 were predicted based on the cryo‐EM structure of the M_4_ receptor in complex with its native agonist acetylcholine and Gi1 (PDB code: 7TRS); [27]. An initial position of clozapine in the orthosteric pocket was obtained by superimposing the complex of a DREADD mutant of M_4_ with *des*‐chloro clozapine bound (PDB code: 8E9X); [28]. Clozapine was obtained by adding a chlorine atom in Maestro (Release 2024–4, Schrödinger LLC, New York, NY, 2024). Minor clashes between clozapine and binding pocket residues in 7TRS.pdb were removed by refining the initial model using the “Refine protein ligand complex” routine of Prime [29, 30] in Maestro. For this purpose, a local optimization of residues found within 5 Å of clozapine was done. As no complex of a muscarinic receptor with an agonist closely related to compound‐6 was available, the binding mode of this compound was predicted “de novo”, using the Induced Fit Docking—Molecular Dynamics (IFD‐MD) approach [31, 32, 33] in Maestro. Two independent runs with different “seed” ligands, which indicate the location of the binding region, were done: 1. *des*‐chloro clozapine from the superposition of 8E9X.pdb on 7TRS.pdb (see above), and 2. the Heptares compound HTL‐9936, as observed in complex with M_1_ (PDB code: 6ZG4, [34]). The membrane setup for IFD‐MD simulations was done based on the membrane embedded M_4_ receptor obtained from the Orientations of Proteins in Membranes (OPM) database (https://opm.phar.umich.edu/, [35]). Models obtained with IFD‐MD were refined with the “refine protein ligand complex” approach of Prime. Qualitatively highly similar binding poses were obtained with both “seed” ligands, showing that the predicted binding orientation of compound‐6 is independent of the IFD‐MD reference ligand. The sequence conservation in the binding regions of compound‐6 and clozapine was analyzed using the Molecular Operating Environment (MOE 2022.02, Chemical Computing Group ULC, 910–1010 Sherbrooke St. W., Montreal, QC H3A 2R7, 2024).

### 2.10 Nomenclature of Targets and Ligands

Key protein targets and ligands in this article are hyperlinked to corresponding entries in http://www.guidetopharmacology.org, the common portal for data from the IUPHAR/BPS Guide to PHARMACOLOGY [36], and are permanently archived in the Concise Guide to PHARMACOLOGY 2019/20 [37].

## 3 Results

### 3.1 cAMP Dynamics Measured in HEK293 Cells Expressing hM_4_ Receptors Reveal Both Inhibitory and Stimulatory G Protein Signaling Pathways

**FIGURE 1:**
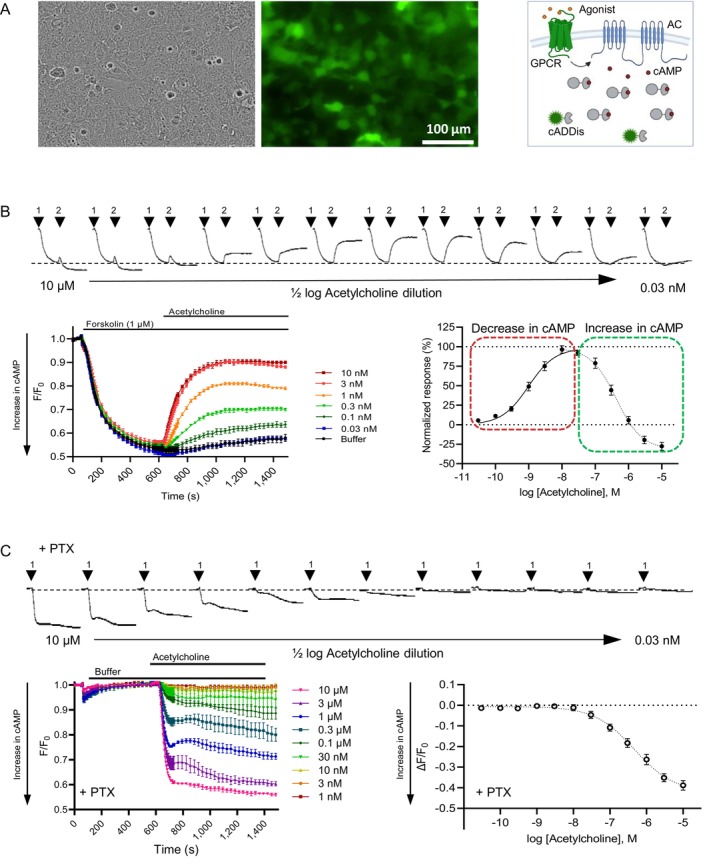
Biphasic cAMP concentration response in HEK‐293 cells expressing human M_4_ receptors. Cells were transduced with a genetically encoded cAMP sensor, and kinetic responses were measured using the FLIPR system. (A) HEK293 cells transduced with cADDis sensors: Brightfield image (left), green fluorescence image (middle), and cADDis assay principle (right) (B) Example kinetic traces for cADDis recordings (each trace represents one well) corresponding to application of different concentrations of acetylcholine. After increasing cAMP and reaching a stable level following forskolin addition (arrow 1, 1 μM, downward signal), acetylcholine (arrow 2) reduced cAMP levels in a concentration‐dependent manner (upward signal) at low concentrations and increased them at higher ones. Average data from multiple wells are combined in a single graph (bottom left panel), and curve fit data of maximum fluorescence changes are plotted as a concentration‐response (bottom right panel). Data are normalized to forskolin control (0%) and maximum response to acetylcholine (100%); pEC_50_ for cAMP decrease was 9.01 ± 0.07, pEC_50_ for cAMP increase was 6.37 ± 0.07 (*n* = 4, 3–4 replicate wells per experiment). (C) Preincubation of M_4_ expressing cells with pertussis toxin (PTX, 0.1 μg mL^−1^, 24 h) blocked the G_i_ specific cAMP decrease but did not alter the increase in cAMP (pEC_50_ = 6.33 ± 0.11; *n* = 2, 3 wells/experiment). All data presented as mean ± SEM.

Investigation of G_i_ coupled receptors is typically performed by measuring the decrease of the second messenger cAMP levels. We achieved high expression levels of the cAMP sensor cADDis in hM_4_‐HEK293 cells by using a commercial BacMam vector for transduction (Figure 1A). The downward version of cADDis, where an increase in cAMP corresponds to a decrease in fluorescence, was used. Adenylyl cyclase (AC) was stimulated by forskolin (1 μM) to increase cAMP levels, reducing the fluorescent signal. The agonist acetylcholine was subsequently added up to a concentration of 10 nM, leading to a concentration‐dependent decrease in the second messenger levels (Figure 1B). Concentrations above 10 nM reversed the fluorescent response, revealing a biphasic concentration‐response curve (CRC), and even stimulated cAMP production beyond forskolin's effect (Figure 1B). The pEC_50_ values for the decrease and increase in cAMP were 9.01 ± 0.07 and 6.37 ± 0.07, respectively. To test whether another G protein is involved, we blocked the G_i_ protein with PTX (0.1 μg mL^−1^) 24 h before the experiment. Results clearly showed that at acetylcholine concentrations above 30 nM, M_4_ activation caused cAMP production in HEK293 cells, independent of G_i_ protein block (Figure 1C). The pEC_50_ value of 6.33 ± 0.11 is similar to the cAMP increase CRC in the experiment without PTX.

### 3.2 M_4_ Receptor Expression Levels in Recombinant Cells Regulate Interaction With Different G Proteins

**FIGURE 2:**
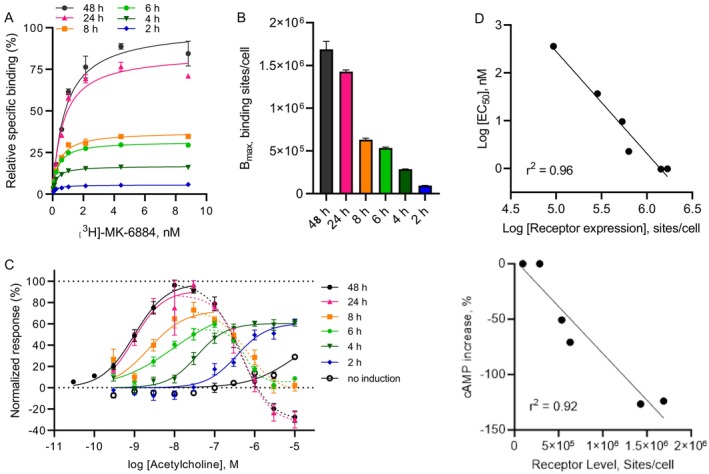
Lower levels of M_4_ receptor expression in HEK‐293 cells alter signaling pathways corresponding to G_i_ and G_s_ protein coupling. Changes in cAMP levels were measured in cells expressing different M_4_ receptor levels. (A) Specific binding of the radioactively labeled M_4_ PAM [^3^H]‐MK‐6884 has been determined in the presence of 100 μM acetylcholine for HEK‐293 cells induced for 2 h, 4 h, 6 h, 8 h, 24 h, and 48 h with a fixed concentration of doxycycline (0.5 μg mL^−1^). With increasing expression time, binding levels increased. Each datapoint represents mean ± SEM of 3 wells. (B) Binding sites/cells ± SEM plotted from (A): 0.94 × 10^5^ ± 0.03 (2 h), 2.85 × 10^5^ ± 0.04 (4 h), 5.34 × 10^5^ ± 0.10 (6 h), 6.30 × 10^5^ ± 0.19 (8 h), 14.2 × 10^5^ ± 0.2 (24 h) and 16.9 × 10^5^ (48 h). (C) cAMP kinetics of cells induced as in (A) using the cADDis cAMP assay. With lower expression levels, the acetylcholine pEC_50_ decreased from 9.01 ± 0.07 at 48 h induction to 6.45 ± 0.11 at 2 h induction for the G_i_ component. It appears that the G_s_ part is absent at 4 h and 2 h induction times (*n* = 2–4, 2–4 wells/experiment). (D) Inverse correlations have been observed between the M_4_ receptor levels and acetylcholine potency (upper panel, for G_i_ signaling), as well as an increase in cAMP at high agonist levels (lower panel, G_s_ signaling).

The physiological relevance of the above changes in the cAMP dynamics is unclear; the effect possibly arising from the artificial nature of the cell line used in this study. Stable cell lines express high protein levels, often much higher than native tissues, which is why, as a next step, we examined the effect of receptor expression levels on the cAMP response to acetylcholine. Human M_4_ receptor expression was induced with doxycycline for varying time periods (2–48 h), and total binding sites were determined by a radioactive binding assay. Using an M_4_ selective radioligand ([^3^H]‐MK‐6884), we found a clear correlation between induction time and receptor expression (Figure 2A,B). Expression levels were 16.9 × 10^5^ ± 0.92 × 10^5^ binding sites/cell at 48 h and 0.94 × 10^5^ ± 0.03 × 10^5^ binding sites/cell at 2 h. We decided to use the [^3^H]‐MK‐6884 instead of the widely used [^3^H]‐N‐Methylscopolamine, which is an unselective muscarinic antagonist, to be able to selectively determine the M_4_ binding sites, in particular for the primary neurons experiment.

Using the same induction protocol, we performed functional cAMP recordings with the cADDis sensor. Data were normalized to the forskolin effect (0%) and the maximum acetylcholine response in 48 h induced cells (100%). Similar results were observed for 48 h and 24 h induction times (Figure 2C). The pEC_50_ for the G_i_ part of the curve was 9.01 ± 0.07 and 9.01 ± 0.20, respectively. Maximal efficacy of ~100% was achieved with ~10 nM and a strong G_s_ effect up to −30% shown at the highest concentration. As induction times shortened, the agonist's potency decreased, with pEC_50_ values for the G_i_ part of the curve of 8.64 ± 0.19 at 8 h, 8.02 ± 0.46 at 6 h, 7.44 ± 0.08 at 4 h, and 6.45 ± 0.11 at 2 h, showing a negative correlation with receptor expression (*r* ^2^ = 0.96, Figure 2D, upper panel). The maximal response was also negatively correlated with total binding sites per cell (*r* ^2^ = 0.92, Figure 2D, lower panel), and cAMP responses decreased up to ~60% for shorter induction times. The G_s_ part of the biphasic curve changed significantly, with cells induced for 4 and 2 h lacking the bimodal profile, indicating that hM_4_ receptor levels affect cAMP signaling after acetylcholine stimulation in multiple dimensions. In the conditions with a strong increase of cAMP at higher concentration, the potential influence of that second phase on the G_i_ E_max_ reported cannot be excluded.

### 3.3 Selective Muscarinic Agonists Show Distinct Functional Profiles Associated With Receptor Expression Levels

**FIGURE 3:**
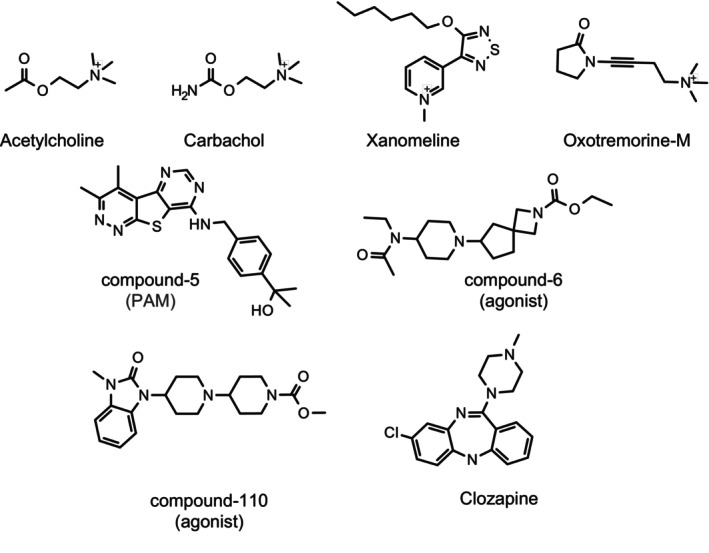
Chemical structures of compounds used in this study. A structurally diverse set of muscarinic agonists was used to probe activation of M_4_ receptors. Compound‐5 (PAM) and compound‐6 (agonist) are arbitrary labeled based on the order they appear in the figure. Compound‐110 has been reported previously (see text for reference).

In order to examine if the specific functional profile observed is unique to the endogenous agonist acetylcholine or relevant to other muscarinic agonists, we conducted an analysis on a structurally diverse set of compounds (Figure 3), which can be practically divided into three distinct groups. Group 1 included acetylcholine and carbachol, which are both small nonselective muscarinic agonists. Group 2 comprised xanomeline and oxotremorine‐M, with a preference for M_1_/M_4_ or M_2_/M_4_ receptors but also active at other mAchR subtypes. Group 3 contained clozapine, compound‐6, and compound‐110, which show high selectivity for M_4_ receptors.

**FIGURE 4:**
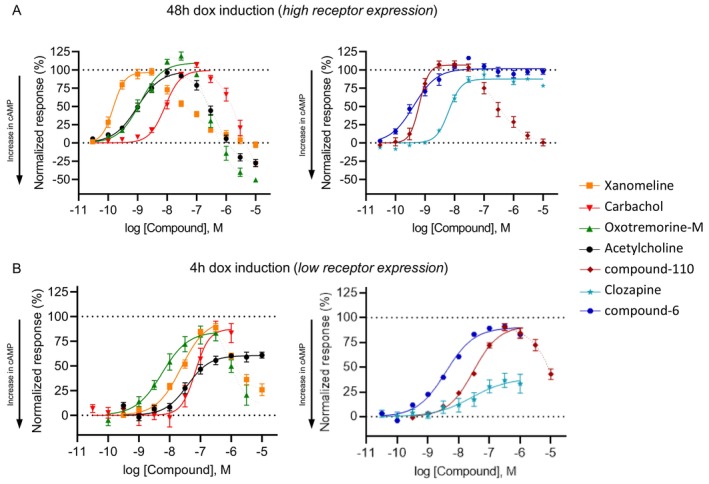
Reduced M_4_ receptor expression shifts cAMP signaling profiles of muscarinic agonists. A set of structurally diverse muscarinic agonists has been tested in the cADDis cAMP assay at high (A) and low receptor expression levels (B). (A) Similar to acetylcholine, the other nonselective agonists carbachol, xanomeline, and oxotremorine‐M showed a biphasic CRC (left). A similar profile is seen for the M_4_ selective compound‐110, whereas clozapine and compound‐6 result in a monophasic curve (right). (B) At lower receptor levels, all curves are shifted to the right, indicating a reduction in potency. Furthermore, the G_s_ part is always less prominent. All agonists show a similar relative efficacy to acetylcholine except clozapine, which appears as a partial agonist at lower expression. All results were normalized to the maximum effect (100%) produced by acetylcholine at either 48 h (A) or 4 h (B). Data presented as mean ± SEM resulted from *n* = 2–4 experiments, 2–6 wells/experiment.

All compounds were tested in the cADDis assay to assess their effect on cAMP levels. With a 48 h induction protocol, oxotremorine‐M, carbachol, and xanomeline exhibited a biphasic CRC similar to acetylcholine with a strong cAMP stimulatory component (Figure 4A). They appeared as full agonists, differing in the G_i_ mode potency: xanomeline was the most potent (pEC_50_ = 9.82 ± 0.06), followed by acetylcholine (9.01 ± 0.07), oxotremorine‐M (8.92 ± 0.07), and carbachol (8.07 ± 0.04). The other three agonists also reached a maximum response of about 100%. Compound‐110 (9.19 ± 0.05) showed a biphasic curve progression. Clozapine and compound‐6 (8.20 ± 0.04 and 9.39 ± 0.06) reduced cAMP to levels similar to acetylcholine up to 10 μM. However, no cAMP increase was observed, indicating a specific interaction with the M_4_ receptor that does not promote engagement of other G proteins other than the G_i_.

Next, we tested the same compounds in cells expressing low receptor levels by inducing hM_4_ expression for only 4 h (Figure 4B). In this case, the data were normalized to the maximum amplitude of the acetylcholine effect measured after induction for 4 h. All agonists showed a decreased potency. Compound‐6 (8.42 ± 0.11) and oxotremorine‐M (8.25 ± 0.13) were the most potent, followed by xanomeline (7.62 ± 0.07), clozapine (7.58 ± 0.45), compound‐110 (7.52 ± 0.06), acetylcholine (7.42 ± 0.08), and carbachol (7.17 ± 0.09) (Table 1). While the G_s_ part disappeared for acetylcholine, it was still present for oxotremorine‐M, xanomeline, and compound‐110, even though not as prominent as with higher receptor expression levels. The selective agonists displayed a change in efficacy: compound‐6 showed slightly lower efficacy than acetylcholine in this condition (90.1% ± 4.47%), whereas clozapine appeared as a clear partial agonist (38.9% ± 9.4%).

**TABLE 1:** Functional profile of muscarinic agonists in HEK‐293 cells and rat cortical neurons expressing human M_4_ receptors.

	HEK‐293, hM_4_, cAMP (48 h)				HEK‐293, hM_4_, cAMP (4 h)						rCN, hM_4_, Ca^2+^ oscillations		
	Top (%)	G_i_ pEC_50_ /slope	G_s_ pEC_50_ /slope	*n* (trials)	Top (%)	G_i_ pEC_50_ /slope	xG_i_ pEC_50_ 48 h	G_s_ pEC_50_/slope	xG_s_ pEC_50_ 48 h	*n* (trials)	Bottom (%)	pEC_50_/slope	*n* (trials)
Acetylcholine	98.25 ± 4.48	9.01 ± 0.07/1.09	6.37 ± 0.07/−1.13	13 (4)	99.25 ± 4.03	7.42 ± 0.08/1.20	0.82	—	—	10 (3)	−1.18 ± 3.53	8.37 ± 0.08/−1.25	3 (1)
Oxotremorine‐M	109.71 ± 4.19	8.92 ± 0.07/1.18	6.51 ± 0.05/−1.35	6 (2)	84.72 ± 6.18	8.25 ± 0.13/1.03	0.93	6.05 ± 0.09/−1.6	0.93	12 (2)	2.86 ± 1.25	7.29 ± 0.03/−1.51	10 (2)
Carbachol	99.32 ± 3.16	8.07 ± 0.04/1.64	5.52 ± 0.14/−1.20	6 (2)	87.35 ± 6.77	7.17 ± 0.09/1.92	0.89	—	—	12 (2)	1.24 ± 11.14	7.19 ± 0.17/−0.84	6 (2)
Xanomeline	96.58 ± 4.12	9.82 ± 0.06/2.23	7.41 ± 0.16/−0.64	6 (2)	96.51 ± 5.03	7.62 ± 0.07/1.08	0.78	6.00 ± 0.09/−2.10	0.81	18 (3)	1.59 ± 1.46	8.29 ± 0.03/1.84	6 (2)
Compound‐110	106.59 ± 3.04	9.19 ± 0.05/2.71	6.63 ± 0.12/−0.90	6 (2)	91.68 ± 3.22	7.52 ± 0.06/1.04	0.82	> 5.00/−0.75	—	18 (3)	−0.97 ± 7.70	7.16 ± 0.09/−1.76	6 (2)
Clozapine	87.43 ± 1.56	8.20 ± 0.04/2.06	—	9 (3)	38.9 ± 9.4	7.58 ± 0.45/0.73	0.92	—	—	22 (4)	—	—	6 (2)
Compound‐6	101.67 ± 2.05	9.39 ± 0.06/1.11	—	6 (2)	90.1 ± 4.47	8.42 ± 0.11/0.94	0.90	—	—	22 (4)	24.89 ± 3.69	8.13 ± 0.11/−1.15	6 (2)

### 3.4 M_4_ Modulation of cAMP Dynamics and Neuronal Activity in Rat Primary Cortical Neurons Resembles Pharmacological Profile Observed in Recombinant Cells

**FIGURE 5:**
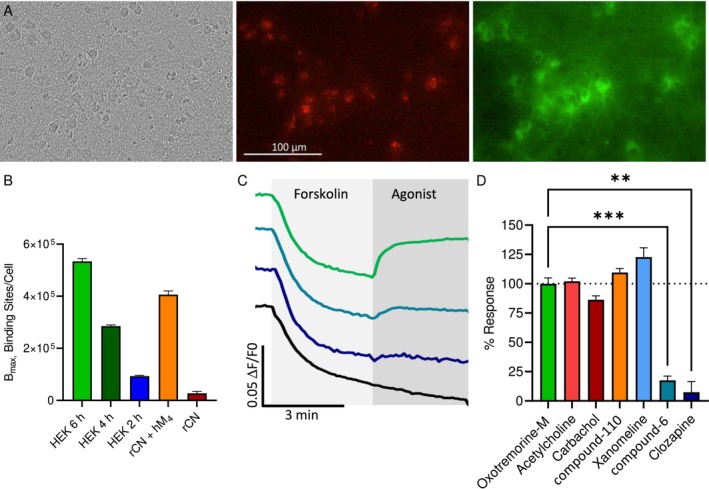
Cyclic AMP recordings in rat primary neurons overexpressing M_4_ receptors. (A) Transduction of rCNs in culture (left panel) with 150 MOI hSyn‐hM_4_‐mCherry‐AAV (middle panel) and 2500 MOI Epac1camps‐AAV (right panel) lead to robust expression after DIV14 (bar = 100 μm). (B) hM_4_ transduced rCNs express low hM_4_ levels (4.06 × 10^5^ binding sites/cells) equivalent to values seen in HEK‐293 cells induced for 4 h and 6 h. Each datapoint represents mean ± SEM of 3 wells. (C) Traces for oxotremorine‐M, compound‐6 and clozapine applied at 10 μM after perfusion with 3 μM forskolin are shown as mean of multiple experiments. (D) The cAMP response to 10 μM agonist is comparable for oxotremorine‐M, acetylcholine, carbachol, compound‐110 and xanomeline but only a small response can be seen upon compound‐6 and clozapine addition. Data presented as mean ± SEM resulted from *n* = 2–5, 2–5 wells/experiment, **p < 0.01, ***p < 0.001; Brown‐Forsythe and Welch ANOVA test, followed by Dunnett's T3 multiple comparisons test with α of 0.05.

In order to assess compound effects on cAMP dynamics driven by M_4_ receptors in a native environment, we used rat primary cortical neurons (rCNs), dissociated from E18 embryos. Based on a published dataset on mRNA expression study (GEO Accession viewer (nih.gov)), the rCNs express only low levels of M_4_ receptors. To obtain M_4_ levels that allow compound testing in the rCNs, we transduced them with an AAV vector containing the human M_4_ receptor and mCherry, allowing assessment of relative transduction efficiency in the red fluorescence channel (150 MOI, titer optimization in Data S1). We measured the effects of the muscarinic agonists on cAMP levels in rCNs by using a second type of a genetically encoded cAMP sensor, EPAC1 [38] that has been cotransduced with M_4_ receptors. The cADDis sensor was not suitable for these experiments as BacMam transduction resulted in significant neuronal toxicity in rCNs. Uniform expression of both mCherry and EPAC1 was observed in most neurons (Figure 5A). Like the cADDis sensor, the EPAC1 sensor, recorded in the green channel, shows a decrease in fluorescence when intracellular cAMP increases. We quantified M_4_ receptor expression in rCNs, with and without viral transduction, using the same radioligand assay as in the hM_4_ stable cell line. Nine days post transduction, we found 0.27 × 10^5^ hM_4_ binding sites/cell in control and 4.06 × 10^5^ sites/cell in AAV‐hM_4_ transduced rCNs (Figure 5B, left panel). These values are comparable to those in hM_4_‐HEK293 cells when expression was induced for 4–6 h (data copied from Figure 2B).

cAMP kinetics of these cells were then recorded using a live cell imaging system, tracking changes in relative fluorescence levels upon perfusion with a buffer containing test compounds. After stimulating cAMP production with 3 μM forskolin, we applied single high concentrations of the test agonist that either caused no effect or a cAMP increase in the HEK system (Figure 5C). By that, we aimed to study whether the compounds showed a similar effect in the neuronal system or still caused the G_i_ induced reduction in cAMP. The maximum response to agonists was quantified as a percentage response relative to oxotremorine‐M (Figure 5D). Agonists oxotremorine‐M, acetylcholine, carbachol, compound‐110, and xanomeline showed a similar response amplitude. However, significantly lower responses were determined for compound‐6 and clozapine treated cells (17.56% ± 3.67% and 7.42% ± 9.16%, Figure 5D). Clozapine, which has also shown reduced efficacy in HEK293 cells induced for 4 h, was even less effective in neurons (< 10%). These findings suggest that both receptor levels and the chosen cellular environment influence the compound's effects.

**FIGURE 6:**
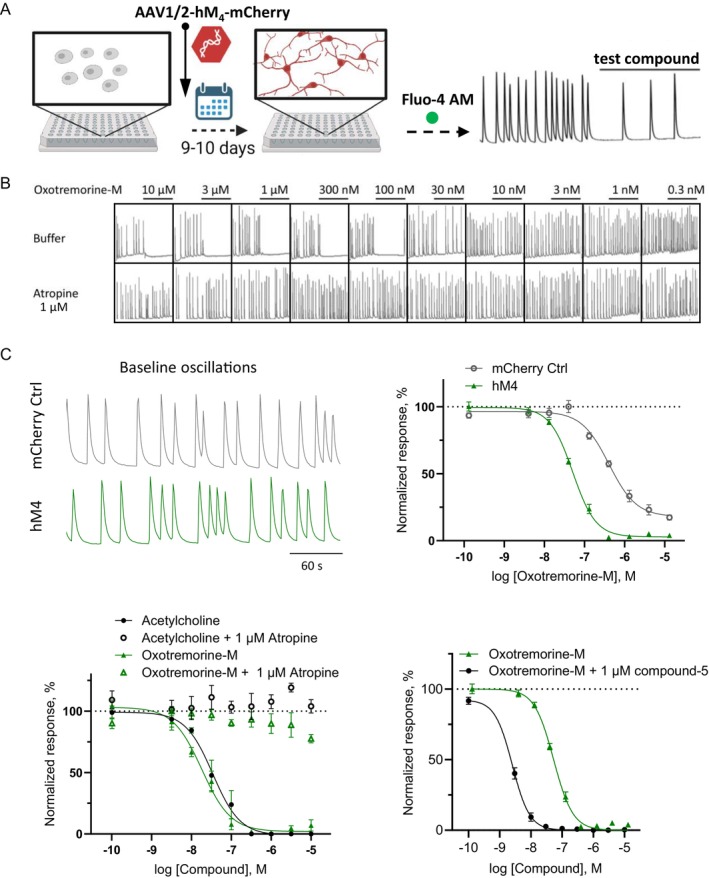
Spontaneous Ca^2+^ oscillations can be used to study the effects of M_4_ receptor activation. The effect of M_4_ receptor‐dependent GIRK channel activation on neuronal activity was measured by assessing the modulation of spontaneous Ca^2+^ oscillations in rCNs transduced with hM_4_ receptors. (A) Protocol used to record Ca^2+^ signals with the Fluo‐4 AM Ca^2+^ dye in rCNs that have been transduced with AAV1/2‐hM_4_‐mCherry to express the M_4_ receptor. Baseline activity is recorded before the addition of a test compound, and changes in oscillation frequency are investigated. Illustration was made with BioRender. (B) Example FLIPR recordings showing spontaneous Ca^2+^ peaks which reduced their frequency upon addition of different concentrations of oxotremorine‐M. Preapplication of atropine prevented inhibition of the Ca^2+^ peaks. (C) Example traces of baseline oscillations of neurons transfected with the mCherry control virus versus the hM_4_ expressing virus (top left panel). In a control experiment, oxotremorine‐M blocked Ca^2+^ oscillations in a concentration‐dependent manner with a higher potency and maximum effect in the hM_4_ expressing neurons (pEC_50_ = 7.29 ± 0.03, E_max_ = 97% block) compared to the control (pEC_50_ = 6.39 ± 0.06, E_max_ = 82% block, top right panel, *n* = 2, 5 wells/experiment). Reduction in oscillations was not present when 1 μM atropine was added, neither in combination with oxotremorine‐M nor with acetylcholine, demonstrating the specific nature of muscarinic activation (bottom left panel). pEC_50_ of the agonists without atropine was 7.74 ± 0.07 for oxotremorine‐M and 7.47 ± 0.08 for acetylcholine (*n* = 1–2, 3 wells/experiment). The effect of oxotremorine‐M was enhanced when applied in combination with the M_4_ selective PAM compound‐5 (1 μM, bottom right panel, *n* = 2, 3‐5wells/experiment).

RCNs in culture provide a system to study not only cAMP levels but also the impact of M_4_ activation on neuronal activity via the GIRK channels signaling pathway. After approximately 1 week in culture, primary neurons form a robust network and begin showing spontaneous activity. This model is useful for studying the effects of various compounds on neuronal firing [24, 26]. To explore the influence of M_4_ activation on network activity, rCNs were plated on DIV0, transduced with AAV‐hM_4_ on DIV1, and used in a FLIPR experiment on DIV9 or DIV10 (Figure 6A). The fluorescent Ca^2+^ sensitive dye Fluo‐4 AM was used to indirectly measure neuronal activity by quantifying the changes in frequency of Ca^2+^ peaks upon application of a test compound (Figure 6B). The activation of G_i_ coupled receptors is known to block neuronal activity through the hyperpolarization of the neuronal membrane potential, a process triggered by the G_i_‐βγ dimer's opening of the GIRK channels [39].

Ca^2+^ oscillations were recorded in control neurons (mCherry control) and in neurons expressing hM_4_ after viral transduction, showing no difference in baseline activity (Figure 6C, top panels). In the radioligand binding studies, it was found that the expression of M_4_ receptors in rat primary neurons is significantly lower (~8 times) compared to neurons after transduction with the human M_4_ AAV virus. In our oscillation assay, the transduction resulted in a more noticeable effect on Ca^2+^ oscillations induced by the muscarinic agonist oxotremorine‐M and led to a ~10 times leftward shift in the corresponding EC_50_ value. This observation suggests that it is the activation of M_4_ receptors that leads to the inhibition of neuronal activity.

The inhibitory effect of oxotremorine‐M was abolished in the presence of the selective muscarinic antagonist atropine (1 μM) demonstrating the specificity of the effect (Figure 6B, bottom traces, and 6C bottom left panel). A similar reduction of Ca^2+^ oscillations was observed with acetylcholine, the effect being also reversed by atropine (Figure 6C, bottom left panel). As acetylcholine activates also nAChRs, this experiment proves that these receptors do not play a role in the inhibition of neuronal activity in this assay.

To further demonstrate the specific involvement of the M_4_ receptor subtype in the modulation of Ca^2+^ oscillations, we combined the agonist application with a highly selective M_4_ PAM, compound‐5. As expected, in the presence of the PAM, oxotremorine‐M showed increased potency, demonstrating the direct and specific contribution of M_4_ receptors (Figure 6C, bottom right panel).

**FIGURE 7:**
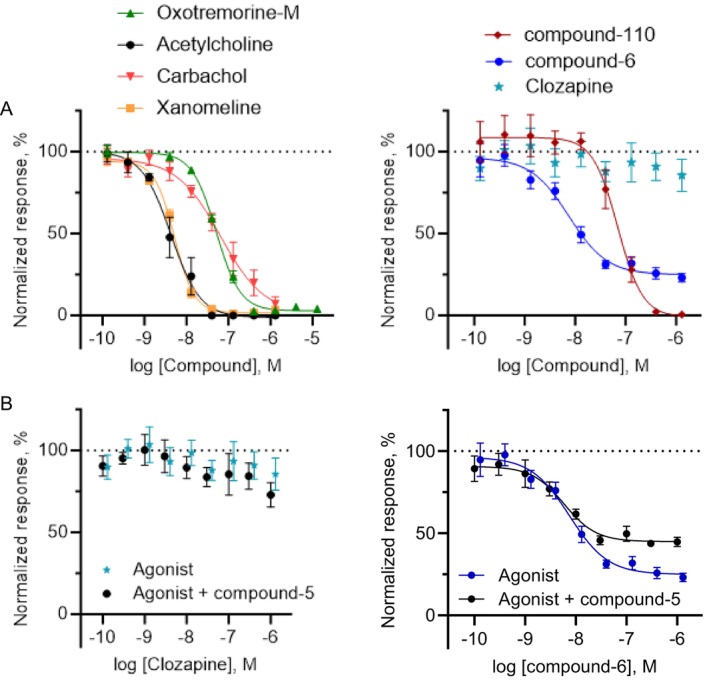
Differential effects of muscarinic agonists on blocking spontaneous neuronal activity. (A) All tested muscarinic agonists fully blocked the neuronal Ca^2+^ oscillations, except for compound‐6, which showed only a partial block, and clozapine, which did not induce any effects. (B) Combined application with compound‐5 (PAM) showed no enhancement of inhibition for clozapine (left panel) or compound‐6 (right panel, *n* = 2, 3–5 wells/experiment).

In order to compare their effect on neuronal activity vs. on cAMP levels, all the compounds used in the hM_4_ HEK‐293 cells study were also tested in the Ca^2+^ oscillation assay (Figure 7 and Table 1). Like oxotremorine‐M, the other pan‐selective agonists induced a full block in spontaneous activity. Compound‐110 showed a similar concentration‐dependent reduction of Ca^2+^ oscillations, causing a full block at high concentrations.

Like in the cAMP assays in HEK293 cells (at low expression levels) and in neurons, compound‐6 and clozapine displayed a diminished maximum effect on blocking oscillations: compound‐6 induced only a partial block (24.89% ± 3.69%), whereas clozapine did not show any effect. When we tested the two agonists in the presence of a PAM (compound‐5), as we did for oxotremorine‐M, no enhancing effect could be observed for clozapine, while for compound‐6 we noticed a decrease in the maximum effect without a change in pEC_50_. Overall, the data on Ca^2+^ oscillations in neurons showed that the tested compounds have a specific profile on M_4_ receptors expressed under a neuronal environment, causing inhibition of neuronal activity, resembling the results seen in the cAMP assays.

### 3.5 Clozapine and Compound‐6 Have Distinct Binding Modes That Influence the PAM Binding Region

The observation that compound‐6 and clozapine are selective M_4_ agonists and that they do not cause an increase of cAMP levels at higher concentrations, as observed for other agonists, motivated us to predict the binding modes of these two compounds in the M_4_ receptor. For clozapine, the recently published structure of a DREADD mutant of M_4_ in complex with des‐chloro clozapine provided a very good starting point [28]. For compound‐6, no such reference experimental structure with a closely related ligand exists, and an unbiased binding mode prediction was necessary (see Methods section for details).

**FIGURE 8:**
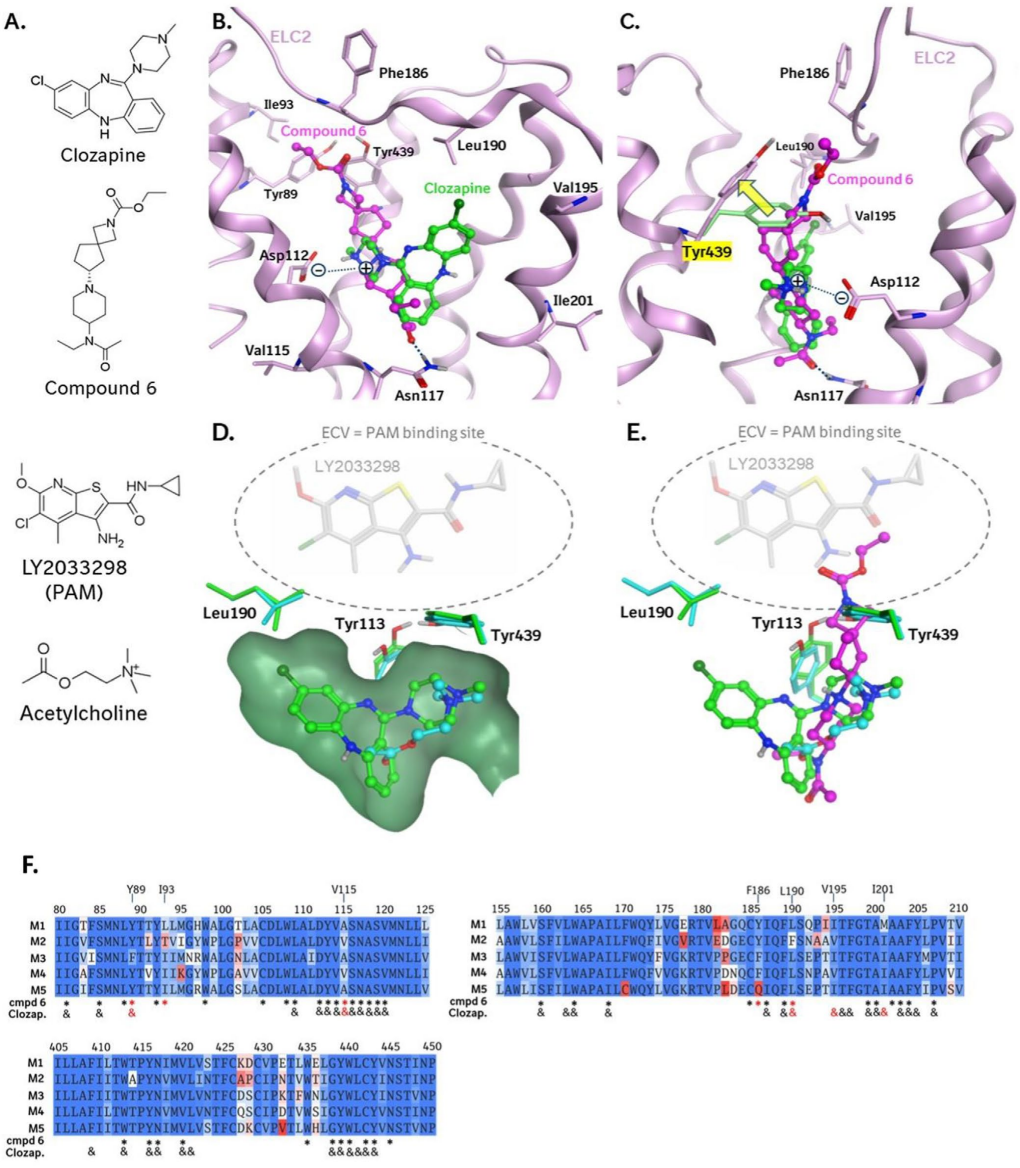
Predicted binding modes of clozapine and compound 6. (A) Chemical structures; (B) Binding model of compound 6 with clozapine superposed. Shown are pocket residues that are either not fully conserved (see sequence alignment) in muscarinic acetylcholine receptors, or that form key ligand‐interactions (Asp 112, Asn 117, Tyr 439). Compound 6 is anchored at the bottom of the orthosteric pocket by an H‐bond to Asn 117. Both compounds have their basic nitrogen atoms in proximity to the negatively charged carboxylate group of Asp 112; (C) Rotated orientation highlighting the movement of Tyr 439 induced by compound 6. This opens the “tyrosin lid” of the orthosteric pocket and allows compound 6 to partially occupy the extracellular vestibule (ECV). (D) Superposition of the cryo‐EM structure of M_4_ in complex with acetylcholine (cyan; [27], PDB code: 7TRS) with the clozapine binding model (green). The positive allosteric modulator (PAM) LY2033298 was superposed from 7TRP.pdb [27] to indicate the PAM binding site in the ECV. Clozapine occupies significantly more space in the orthosteric binding pocket (surface shown) than acetylcholine. It packs tightly against residues that contribute to the PAM binding pocket (Leu 190, Tyr 113, Tyr 439), influencing their conformational properties. (E) Same as (D), without the surface of the orthosteric pocket, making the clozapine‐induced movement of Tyr 113 more obvious. Compound 6 is superposed from our binding model to show that it partially occupies the PAM binding site. F. Segments of the sequence alignments of the M_1–5_ receptors that contain residues located within 6 Å from compound‐6 (*) or clozapine (&) in the predicted binding complexes with M_4_. Nonconserved M_4_ residues are specified and highlighted by red symbols (*, &).

Our predictions show highly different binding modes for clozapine and compound‐6 (Figure 8): Clozapine is predicted to maintain the reported binding mode of its des‐chloro derivative [28]. Its rigid molecular scaffold tightly fills the available space in the orthosteric agonist pocket. However, some, albeit limited, structural changes are necessary to accommodate the large ligand, in comparison to the reference structure with acetylcholine bound to M_4_ (7TRS.pdb, Figure 8D,E). Most strongly affected is the side chain of Tyr 113, which is part of the “tyrosine lid” on the orthosteric pocket, that at the same time forms the bottom of the extracellular vestibule (ECV), the binding location of PAMs. The contact with the piperazine ring of clozapine somewhat displaces this residue such that the position of its hydroxyl group moves in the direction of the PAM binding region by 1.5 Å. Another residue contributing to the ECV PAM binding site is Leu 190. The chlorine atom of clozapine contacts this residue and induces a minor change in its side chain orientation.

In our induced fit docking simulations for compound‐6, the dimensions of this compound turned out to be incompatible with those of the orthosteric pocket alone. Compound‐6 is predicted to induce a conformational change of the side chain of Tyr 439. This allows its 5‐4‐spiral ring system to protrude from the orthosteric pocket into the ECV. This spiral system and the terminal carbamate group are predicted to partially occupy the ECV region (Figure 8E).

For residues found within 6 Å from the two ligands, we analyzed the sequence conservation of M_1‐5_ (Figure 8F). Five nonconserved residues are found in the two environments, three of which are common to both ligands (Tyr 89, Val 115, Leu 190). However, none of these residues are characteristic for M_4_ and can thus explain the functional selectivity of the two ligands for this receptor in a straightforward way. For clozapine, the combination of Leu 190 and Val 195 is found only in M_4_. No such characteristic pair of neighboring residues was identified for compound 6.

## 4 Discussion

In this study, we investigated the pharmacology of the M_4_ receptor, focusing on the modulation of intracellular cAMP levels and GIRK channel activation pathways. We observed that agonist concentration and receptor expression influenced changes in downstream signaling between the inhibitory and stimulatory cAMP pathways. From a structurally diverse set of muscarinic receptor agonists, we identified specific signaling profiles for different compounds. Notably, two compounds with high selectivity for M_4_ receptors over other muscarinic subtypes specifically engaged the inhibitory cAMP signaling pathway and demonstrated reduced efficacy in inhibiting spontaneous network activity in primary neurons.

The M_4_ receptors, acting as G_i_ coupled GPCRs, inhibit adenylyl cyclase via the Gα subunit of the G_i_ protein, reducing cAMP levels. This mechanism, typically studied for M_4_ activation, has been investigated in the past by using [^35^S]‐GTPγS and/or a Ca^2+^ mobilization assay [40, 41, 42, 43, 44]. Whereas these assays do not directly report cAMP levels, the cADDis assay used in our study allows direct measurement by a fluorescent sensor [45] as well as the kinetics of the response. It showed acetylcholine to be more potent than in previous assays [42, 44], with a unique biphasic profile at different concentrations. This profile was not reported in literature using the assays mentioned above. The mismatch between assays could be due to their differing principles: the [^35^S]‐GTPγS measures G protein activation without distinguishing between G_i_ and G_s_ coupling, while the Ca^2+^ mobilization assay uses an artificial system (transfection of a chimeric G_i_ protein) that can mask the binding of the endogenous G protein. These differences can also account for the observed differences in agonist potency.

There are also reports that used either [^3^H]‐cAMP or gene reporter assays and observed a biphasic cAMP modulation profile for the agonist carbachol in M_4_ expressing cell lines. They reported an increase in cAMP at higher agonist concentrations and hence the potential involvement of another G protein besides G_i_ in M_4_ signaling [46, 47]. We observed in our study a large shift between the activation of the G_i_ and G_s_ pathways (see pEC_50_ data in Table 1) for the agonists that showed a biphasic profile. Figueroa et al. [18] reported high concentrations required for the G_s_ effect as well, suggesting that this effect might be physiologically irrelevant.

By completely blocking the G_i_ protein by PTX, we demonstrated that the response measured at low acetylcholine concentrations, which corresponds to the decrease in cAMP levels, is driven by this G protein type. The increase in cAMP, at higher agonist concentrations, we assume to be caused by coupling to G_s_ protein, as it remained unaffected following treatment with PTX, similar to what was shown by McDonald et al. [19]. Previous studies using a similar cellular system as ours ruled out G_q_ effects of other muscarinic receptors that might have influenced the observed effect.

In our cellular model, we employed a protocol for M_4_ receptor expression regulation, based on the Tet‐On system. To enhance reliability, a strategy of time‐dependent incubation with doxycycline was incorporated. We showed a correlation between doxycycline incubation time and M_4_ expression using a radioligand binding assay. Changes in receptor expression showed a direct impact on cAMP responses, altering pEC_50_ values and reducing stimulatory effects on cAMP generation. Prior studies, including [46, 48], reported that the effect is dependent on receptor expression levels, similar to our findings. The mechanism behind this is disputed. Newman‐Tancredi et al. [49] suggested a G protein switch due to dimerized receptor formation at high expression levels. Another theory, based on Michal et al.'s [48] work, suggests G_i_ protein depletion could cause G_s_ protein binding. Our results showed a stimulatory cAMP effect already at lower receptor densities, indicating available G_i_ proteins for a larger inhibitory response.

This study's strength also lies in the investigation of diverse agonists' effects on the unusual signaling profile, allowing us to identify differences in cAMP regulation. In HEK‐293 cells, five out of seven tested compounds showed a biphasic response similar to acetylcholine. Clozapine and compound‐6 only reduced cAMP levels with no G_s_ coupling up to 10 μM. This is often described as biased signaling for GPCRs [50]. Specific ligand‐dependent receptor conformation can recruit different G protein families, leading to multiple or single signaling pathways [16] as seen with clozapine and compound‐6. The complexity of quantifying bias in G_i_ vs. G_s_ signaling pathways often presents a significant challenge. While we have assigned G_i_ and G_s_ EC_50_ values, the competitive nature of these pathways complicates direct quantification of bias. The overlapping responses of G_i_ and G_s_ signaling make it difficult to isolate the potency of ligands for each pathway independently. Our observations align with the guidelines for GPCR ligand bias provided by the International Union of Basic and Clinical Pharmacology [51], even if precise quantification is not feasible. To address these limitations, future experimental approaches could include using PTx to inhibit G_i_ signaling and cholera toxin (CTx) to inhibit G_s_ signaling, employing genetic knockout models to study their individual contributions, and utilizing advanced quantitative methods like bioluminescence resonance energy transfer assays (BRET) and tagged G proteins to gain more precise insights into signaling pathways.

Not only receptor density and ligand type influence GPCR signaling, but also the cellular type determines downstream pathways [16]. Our experiments in neurons showed most agonists signaling only through cAMP inhibition. When tested at 10 μM concentration, all compounds showed cAMP modulation in the inhibitory direction, without any hint of cAMP stimulatory effect observed in the cell. Clozapine and compound‐6 had a slightly different profile in neurons, still pointing to inhibition of cAMP levels, but with a very partial effect, even though they appeared as full agonists in the HEK293 cells. Randáková et al. [52] observed a biphasic curve in CHO cells and rat brain tissue, similar to our findings in HEK‐293 cells. The brain tissues had 10 times lower muscarinic receptor expression than CHO cells, suggesting heterologous expression system findings could translate to the native environment.

One limitation of the functional cAMP recordings in primary neurons is that we tested only a single drug concentration. We cannot exclude the possibility that increasing the concentration of the agonist might reveal a stimulatory effect. Conversely, reducing the agonist concentration could potentially enhance the inhibitory effects of clozapine and compound‐6, which were only partial at the tested concentration. However, the cAMP data, despite being limited to a single concentration, should be considered alongside the effects of M_4_ activation on neuronal activity, which were evaluated across a full range of concentrations. As previously mentioned, G_i_ coupling is associated with reduced neuronal activity in this system. Both compounds demonstrated a concentration‐dependent response in the oscillations, with partial effects observed even at 10 μM, aligning well with the cAMP recordings.

G_i_ protein‐coupled GPCRs activate also GIRK channels, a family of potassium channels that induce hyperpolarization of the membrane potential and reduction of neuronal excitability. Using the same method previously applied for studying metabotropic glutamate receptors' pharmacology in rCNs [26, 53], we examined M_4_ activation's impact on neuronal activity, measured as spontaneous Ca^2+^ oscillations. All agonists with full efficacy in the EPAC1 cAMP assay and dual G_i_/G_s_ coupling in the cADDis assay blocked neuronal spontaneous Ca^2+^ oscillations. The pEC_50_ values for neuronal activity modulation align well with cAMP generation inhibition in HEK‐293 cells, especially after the 4 h induction interval (low M_4_ expression). As expected, we did not observe a bimodal effect on neuronal activity, as GIRK channel activation is typically associated only with βγ subunits of the G_i_ protein. Clozapine and compound‐6, which selectively caused cAMP inhibition in the HEK‐293 assay and partially inhibited cAMP in neurons, had a different profile on Ca^2+^ oscillation modulation. Clozapine was inactive, while compound‐6 partially reduced Ca^2+^ oscillations. Attempting to enhance the orthosteric agonists' activity using a selective M_4_ PAM (compound‐5) and by that to shift its potency to lower concentrations, as seen with oxotremorine‐M, failed in combination with clozapine and compound‐6. The lack of potentiation for the two agonists could also result from probe dependency often seen with allosteric modulators.

Our binding mode predictions for compound‐6 and clozapine show highly different binding modes (Figure 8). However, both compounds show some impact on the ECV PAM binding region. Compound‐6 occupies part of this allosteric pocket, and clozapine packs tightly against residues that contribute to it, leading to conformational changes in the side chains of Leu 190 and Tyr 113. It is expected that the tight packing of clozapine against Tyr 113, Tyr 439, and Leu 190 will strongly influence the mobility of these residues, which are important contributors to the ECV PAM binding site. These observations indicate that clozapine also influences the shape of the ECV PAM binding site. We propose that these interferences of clozapine and compound‐6 with the ECV PAM binding site affect the binding of compound‐5 and thus explain why this PAM had no effect on the activity of these two agonists in primary neurons.

While the observations might resemble GPCR biased signaling, it is not straightforward to precisely describe the mechanism. Upon receptor activation, G_iα_ dissociates from G_iβɣ_, modulating cAMP levels through adenylate cyclase and activating GIRK channels via G_iβɣ_ subunits. Thus, postactivation, subunits can engage either pathway. A simpler explanation could be that compounds like clozapine and compound‐6, displaying partial activity at lower receptor expression in both HEK‐293 cells and primary neurons, reduce cAMP (a highly amplified signaling cascade) but fail to activate enough GIRK channels for sufficient membrane hyperpolarization to modulate neuronal activity. But how can this be linked to structural differences between these different agonists?

There is another characteristic of clozapine that must be mentioned here. Clozapine is a selective functional M_4_ agonist that also binds to the orthosteric site of the other muscarinic receptors (M_1_, M_2_, M_3_ and M_5_) but acts as an antagonist [54]. This could also be the case for compound‐6. Compound‐6 shows some similarity to HTL9936, which has been disclosed previously as a selective M_1_ receptor agonist, with reduced activity at the other muscarinic receptor subtypes [34, 55]. Considering that the sequence of the orthosteric site of all five subtypes of muscarinic receptors is highly conserved (100% for residues found within 5.0 Å of acetylcholine) we can hypothesize that these two compounds must engage a structural motif in the M_4_ receptors that is specific to this subtype. This would be possible if the compounds also interact with the receptor pocket that is part of the less conserved allosteric site, as this might explain both the subtype selectivity observed for the two compounds and the particular signaling reported in this study. This is, in fact, one of the conclusions derived from the binding mode predictions: both compounds appear to interfere with the allosteric site positioned just above the orthosteric site. The sequence alignment (Figure 8) shows that some nonconserved residues can be found in proximity to clozapine and compound‐6. However, based on our static binding models, it is not straightforward to connect these sequence variations to subtype or functional selectivity. Potentially, such nonconserved residues are involved in conformational changes of the receptor that are relevant for activation and signaling, and their role will only become obvious when the dynamics of activation and G‐protein binding are analyzed.

Other reports confirm clozapine's specific pharmacology. Olianas et al. [56] found that clozapine inhibited cAMP production in CHO cells expressing human M_4_ but not in rat striatal membranes. Intriguingly, it acted as a competitive antagonist in rat striatal membranes when coapplied with acetylcholine. Clozapine's indirect antagonistic action was also seen in CHO cells, appearing as a partial agonist, reducing carbachol's overall receptor activation effect [48, 57]. Among all pan‐muscarinic agonists used in this study, clozapine's action varied with receptor expression: full agonism on cAMP modulation at high M_4_ levels, partial agonism at low M_4_ levels, partial effect in primary neurons, and no effect on neuronal activity modulation.

Partial agonists, such as clozapine and compound‐6, offer both advantages and disadvantages in terms of therapeutic potential. On one hand, their selectivity and partial activity can lead to reduced activation in peripheral tissues, thus minimizing adverse events. On the other hand, these compounds may not fully activate targeted receptors, particularly in tissues with low expression levels, as observed for M_4_ receptors in the hippocampus and cortex. This could potentially affect their efficacy in patients with cognitive impairment, primarily associated with pathological alterations in the hippocampus or cortex. However, this may be less critical for their effectiveness in addressing psychosis in schizophrenia, as those symptoms are driven by brain circuits in the striatum, where M_4_ receptor is highly expressed.

In conclusion, our study offers valuable insights into the M_4_ receptor pharmacology, particularly on the modulation of intracellular cAMP levels and activation of GIRK channel pathways. We have unveiled the distinct signaling profiles of various muscarinic receptor agonists, underscoring the complexity of M_4_ receptor pharmacology. These findings have significant implications for drug discovery, particularly for the development of novel M_4_ muscarinic modulators. Understanding the dual signaling of receptor activation can guide the development of new drugs with better efficacy and reduced side effects profiles. Partial agonists like clozapine and compound‐6 present both benefits and drawbacks, with their selectivity and partial activity potentially reducing adverse events, but possibly affecting efficacy in tissues with low receptor expression. Future research should further explore these mechanisms, investigate their structural determinants and their therapeutic implications, and utilize this knowledge in designing improved M_4_ muscarinic modulators.

## Author Contributions

M.M., C.P., and M.P. conducted the in vitro studies. O.H. was responsible for the molecular modeling. U.G. oversaw the compound selection and synthesis. D.U. supervised the studies and, together with M.M. and O.H., drafted the manuscript. All authors read, edited, and approved the final manuscript. At the time the study was conducted, all authors were employed by Boehringer Ingelheim (C.P. was on an industrial student placement from the University of Manchester, UK).

## Ethics Statement

All animal research was conducted according to German Animal Protection Law (§4 Abs. 3 TierSchG, experiment number: 18‐015‐O) and with local animal care guidelines and the Association for Assessment and Accreditation of Laboratory Animal Care (AAALAC) regulations, the German Animal Protection Law (Tierschutzgesetz), and the EU Directive 2010/63/EU, as well as the United States Department of Agriculture Animal Welfare Act. All experimental studies were performed in an AAALAC certified facility.

## Conflicts of Interest

M.M., M.P., O.H., U.G., and D.U. are employees of Boehringer Ingelheim Pharma GmbH & Co. KG. All other authors declare no potential conflicts of interest.

## Supporting information


Data S1.

## Data Availability

All data generated and analyzed during this study are included in this article. Numeric source data for the presented figures are available from the corresponding authors on reasonable request at the desired level of analysis.
