# Supplementary figures and images for: Downstream Signaling of Muscarinic M4 Receptors Is Regulated by Receptor Density and Cellular Environment
Source: Pharmacol Res Perspect. 2025 May 19;13(3):e70123. doi: 10.1002/prp2.70123 (PMC12086361; doi:10.1002/prp2.70123)

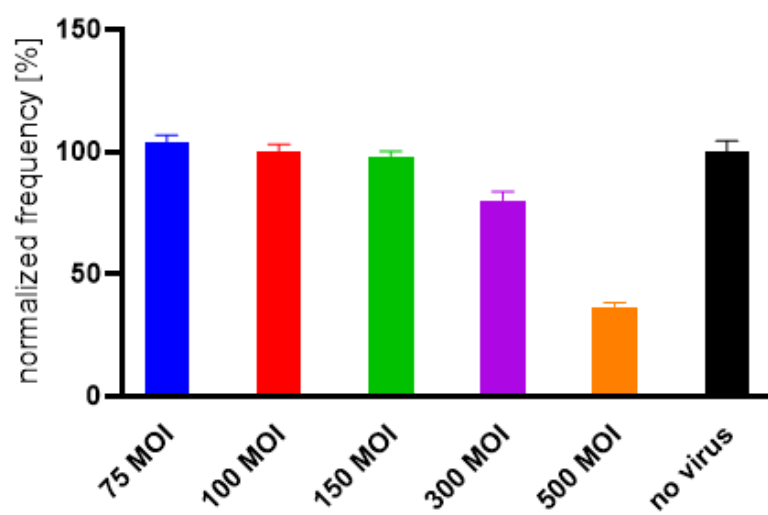

**Supplemental 1**

Supplement: Supplementary file 1 — Data S1. [file PRP2-13-e70123-s001.pdf]
